# The Deciphered Genome of *Mesobuthus martensii* Uncovers the Resistance Mysteries of Scorpion to Its Own Venom and Toxins at the Ion Channel Level

**DOI:** 10.3390/toxins5112209

**Published:** 2013-11-18

**Authors:** Nicolas Andreotti, Jean-Marc Sabatier

**Affiliations:** Laboratoire INSERM UMR 1097, Université d’Aix-Marseille, 163, Avenue de Luminy, Case 939, TPR2 INSERM, 13288 Marseille Cedex 09, France; E-Mail: andreotti.nicolas@gmail.com

Scorpions are amongst the most ancient arthropods, as the oldest fossils were dated from the late silurian (*Proscorpius osborni*, 418 million years B.P.) [1]. Extant scorpions (with about 2,000 species spreading globally) are therefore ‘living fossils’, which retained all of the primitive features except that they are considerably smaller compared to their up to 1-meter long *Brontoscorpio anglicus* ancestor (a species of fossil scorpion from Silurian to lower Devonian) [2]. From the fossils of the late Silurian period, scorpions were found to have developed and adapted their venomous systems [1]. Evolving over the past 400 million years, scorpion venoms contain a variety of compounds, including the more or less potent and selective peptide toxins acting on the diverse and ubiquitous ion channel proteins. The latter are widely distributed in many systems, such as nervous, skeletal and cardiovascular in the scorpion’s prey (or predator). Basically, the severity of scorpion envenomation ranges from local pain and paresthesia to lethal cardiotoxicity and encephalopathy [3]. At present, it is a serious cause of mortality in several Latin countries, in South America, southern and western Asia, and Africa. The number of scorpion stings is estimated to be *ca*. 1.2 million per year worldwide, resulting in more than 3,200 deaths [4]. Scorpions are known for their deadly venoms but are likely resistant to their own venoms due to the ‘sexual’ stings and cannibalism occurring in many scorpion species. In 1998, it was reported that the scorpion *Androctonus australis*, as well as its muscle and nerve fibers, were insensitive to both its own venom and purified toxins [5]. Fifteen years later, the mysterious veil of scorpion resistance to its own venom and toxins was unmasked at the molecular level, by the deciphered draft genome sequence of the Chinese golden scorpion *Mesobuthus martensii* (eastern Asia) that has been recently published by the research group of Professor Wenxin Li [6]. The project started at Wuhan University back in the 1990s, when Professor Li and his colleagues continuously discovered scorpion toxin cDNAs after constructing the first venomous gland cDNA library of *M. martensii* [7]. They pursued in exploring scorpion toxin diversity and the resistance mechanism of scorpion to its venom. The deciphering of *M. martensii* draft genome sequence is remarkable in several aspects. First, this genome contains about 32,016 protein-coding genes, whereas humans ‘only’ have about 22,500 genes (International Human Genome Sequencing Consortium, 2004). The scorpions exhibit an arsenal of *ca*. 160 enzymes to allow detoxifying harmful plant chemicals (from insects eaten) and digesting fats. Some enzymes also chemically modify coumarin into some fluorescent derivatives to make scorpions ‘fluorescent’ when exposed to UV light. Second, it completely describes the molecular diversity of scorpion toxin genes [6]. Indeed, the authors highlighted a total of 116 neurotoxin genes (of which 45 were unknown), consisting of 61 sodium channel, 46 potassium channel, 5 chloride channel, and 4 calcium channel toxin genes. Third, Cao and collaborators successfully cloned two functional scorpion potassium channel genes [6]. The two genes encoding K^+^ channels from *M. martensii*, MmKv1 and MmKv2, correspond to voltage-dependent ion channels, sharing homology with the mouse voltage-gated Kv1.3 potassium channel. After obtaining scorpion potassium channel genes, they expressed MmKv1 and MmKv2 channels in HEK 293 cells, and used ‘classic’ small blockers to efficiently inhibit the scorpion K^+^ channel currents (a first investigation of ion channel currents from different scorpion ion channel subtypes). Interestingly, the authors further showed that the two functional scorpion K^+^ channels were almost insensitive to the related scorpion venom, as well as to charybdotoxin, a scorpion blocker of several potassium channel subtypes, including the Kv1.3 channel. Accordingly, the inhibition of *M. martensii* venom toward both K^+^ channels, MmKv1 and MmKv2, was about 100-fold lower than that of the mouse Kv1.3 channel. Structurally, it was pointed out that one of the critical amino acid residues near the selectivity filter region was changed to arginyl in MmKv1 channel, and to lysyl in MmKv2 channel. Such amino acid residue changes are thought to confer some ‘immunity’ of scorpion potassium channels to scorpion toxins, as shown previously in mammalian and non-mammalian K^+^ channels [8,9]. Hence, two scorpion potassium channels (MmKv1 and MmKv2) for the first time illustrated one important mechanism that provides scorpion with resistance to its venom and neurotoxins, implying some co-evolution of ion channels with neurotoxin genes in *M. martensii* scorpion. Fourth, the deciphered genome of *M. martensii* paves a ‘novel’ way to research scorpion ion channels. Indeed, the genome of *M. martensii* basically provides much valuable sequence information to clone, identify and characterize more scorpion ion channels from now on. Together with the newly discovered *M. martensii* scorpion toxins with various ion channel selectivities, upcoming structure–function relationship studies on both scorpion ion channel types/subtypes and toxins, will undoubtedly help to dissect and/or unravel the remaining mysteries of scorpion at a molecular level.
